# Respiratory symptoms amongst females in a fishing settlement in the Niger Delta, Nigeria

**DOI:** 10.4102/phcfm.v3i1.152

**Published:** 2011-02-22

**Authors:** Alexander B. Akani, Paul O. Dienye, Ita B. Okokon

**Affiliations:** 1 Department of Family Medicine, University of Port Harcourt Teaching Hospital, Nigeria; 2 Department of Family Medicine, College of Medical Sciences, University of Calabar, Nigeria

## Abstract

**Background:**

Approximately half of the earth's population in the rural areas of developing countries uses energy obtained from biomass burning, which is harmful to people.

**Objectives:**

This study is aimed at determining which respiratory symptoms can be associated with biomass burning amongst fish smokers in the Oyorokoto fishing settlement.

**Method:**

A community-based, cross-sectional questionnaire, which employed a modified cluster sampling technique, was used.

**Results:**

A total of 300 subjects were recruited for the study, of which 210 (70%) were fish smokers. The mean age was 31.46 ± 13.03 years, with the majority (42.0%) having only primary school education. The prevalence of respiratory symptoms amongst the subjects was 86.7%, the most frequent of which were catarrh (30.48%) and a cough (28.57%). The respiratory symptom occurring least frequently was breathlessness (2.38%). The symptoms most often experienced during fish smoking were those of catarrh (75.5%) and sneezing (73.0%), whereas breathlessness occurred the least, in only 7 (3.3%) of the participants. Sneezing stopped in 64.2% of the subjects after fish smoking had ceased. Most of the fish smoking took place indoors.

**Conclusion:**

Health promotion featuring preventive interventions, such as the wearing of face-masks and the use of modern fish smoking methods, which is associated with fewer health risks, is essential to improving the quality of life of fish smokers. The government's provision of certain social services, including better education opportunities for the young, is advocated, and should be especially targeted at improving the lot of the girl child.

## Introduction

Wood and other forms of biomass are the largest source of domestic energy in developing countries.^1, 2^ Approximately half of the earth's population and over 90% of households in the rural areas of developing countries use the energy which they gain from burning biomass, such as wood, charcoal, animal excrement and agricultural residues.^2^ Biomass burning tends to result in a high level of indoor air pollution, which has an important global impact on mortality and morbidity rates.^1, 2, 3^ The burning of biomass consists of three stages: (1) ignition, (2) flaming (burning and smoking with flame) and (3) smouldering (burning and smoking without flame). Biomass burning is the main source of toxic gas, particulate matter and greenhouse gases on the planet.^4^ Not only does it affect the physical and chemical makeup of the atmosphere, but it also produces chemicals which alter the pH of rainwater significantly,^5^ affecting the thermal balance of the atmosphere by restricting the amount of solar radiation that is reflected to outer space.^6^

According to the World Health Organisation (WHO),^7^ about 2.8 million people die annually from indoor air pollution worldwide, translating to about 6% per year of all deaths worldwide. Of such deaths, 2.44 million occur in the developing countries, including Nigeria.^7^ Developing countries also show higher rates of chronic respiratory diseases, including chronic obstructive pulmonary disease (COPD), amongst those populations which are exposed to indoor cooking smoke, rather than to tobacco or atmospheric pollution. By comparison, in developed countries, the commonest cause of COPD has been identified as tobacco smoking.^8, 9^ Amongst the reported respiratory effects of chronic exposure to wood smoke and other forms of biomass burning in adults from developing countries, an increased prevalence of chronic bronchitis has been found.^10^ This increased prevalence was observed amongst Nigerian soldiers who were exposed to domestic biomass fuel combustion.^11^ Johnson and Aderele^12^ have also reported an association between indoor smoke pollution and bronchopneumonia, or acute respiratory infection (ARl). In accordance with such findings, pollution has been found to contribute to the aetiology of ARIs which appear to be a major killer of children, accounting for the deaths of from 4 to 5 million pre-school children per year in developing countries.^13^ In a study which was conducted in Lagos by Femi-Pearse et al,^14^ a statistically significant association was observed between the use of wood fire and persistent morning phlegm (*p* < 0.05). Other respiratory symptoms found to be associated with the inhalation of wood smoke during fish smoking include a cough with or without sputum, haemoptysis, dyspnoea, a runny nose, sneezing and a decline in the lung function indices, apart from FEV1 percentage.^15^ To our knowledge, no field study had, prior to this study, investigated the respiratory symptoms associated with biomass burning among the fish smokers in Oyorokoto, a fishing settlement area in the Niger Delta region, in which fish smoking is an important occupation. This study, therefore, was undertaken to determine the magnitude of this problem, in order to see what health promotion efforts should be made to minimise the development of such symptoms and their long-term effects.

## Ethical considerations

The study employed a community-based, cross-sectional questionnaire, which was approved by the Ethical Committee of the University of Port Harcourt Teaching Hospital. Before commencement of interviews, the objectives of the study and the contents of the questionnaire were explained to each of the subjects by research assistants, and consent for participation obtained. Participants were assured that the data which was gathered would be used only for research purposes.

## Method

Research was carried out in the Oyorokoto fishing settlement in the Andoni local government area of the Rivers State. The fishing settlement is the largest in West Africa, and is located at the tip of an offshore island in the Atlantic Ocean. The inhabitants of the island are predominantly fish farmers, while a few work as traders, artisans, clergymen and civil servants in the local government council office in the community. The island has a population of over 10 000, of which more than 50% are women.16 The local community is rural, and is not supplied with electricity, pipe-borne water or standard health facilities. The settlement is within reach of Port Harcourt, the Rivers State capital, by means of a one-and-a-half hour trip in a speedboat. The sample population consisted only of women, as fish smoking, traditionally, is considered to be a fitting occupation for women in the community. Male involvement in such domestic activities as fish smoking is prohibited by the patriarchal nature of the society. Only women aged between 15 years and 64 years, who had resided in Oyorokoto for at least 12 consecutive months prior to the study, and who had given their consent after the implications of the research had been carefully explained to them, were included in the study.

Those who had been involved in activities such as pipe or cigarette smoking, which predisposed them to the development of respiratory symptoms, and those who refused to give their consent to participate in the research, were excluded from the study. A sample size of 384 was calculated, using the formula *N* = Z^2^ (*p*) (q)/d^2^. The sample size was adjusted to 300, considering the fact that the female population of Oyorokoto was less than 10 000, using the following formula:

*n*_f_ = *n*/1 + (*n*)/(*N*),

where

*n*_f_ = the desired sample size with population less than 10 000; *n* = the desired sample size with population more than 10 000; and *N* = the estimated population size.

The commonly used two-stage ‘30 × 7’ cluster sampling scheme was employed. The WHO developed this method with the aim of calculating the prevalence of immunised children within ± 10 percentage points of the true proportion, with 95% confidence. In this context, if the true prevalence were to be 40%, one would expect an estimate of between 30% and 50%, with the use of the ‘30 × 7’ method. This sampling scheme is considered adequate for the sampling of most community health factors, and has been adopted for other purposes such as rapid needs assessments, with no (or only slight) modification.^17, 18, 19^

In this study, based upon 2006 Nigerian census tract data,^16^ 125 enumeration areas in the community were identified as relevant clusters, and of these, 30 were randomly selected. The residential addresses of households within the selected enumeration areas were used to create a new sampling frame, with 10 addresses randomly selected for each of areas concerned. The selected households represented a cluster, from which data regarding respiratory symptoms were collected. In all, 300 houses were sampled, from which the participants were selected.

Ten volunteers were recruited as research assistants from amongst the community health extension workers in the local government, and received training over 3 days on the research process.

Participants were asked about the definite respiratory symptoms being assessed, such as sneezing, catarrh (nasal discharge), a cough, phlegm, breathlessness and chest pain. They were also asked which of the symptoms occurred during fish smoking, and which of these stopped after fish smoking. The information obtained from the subjects was recorded on the questionnaires, which were then checked for any missing data and the latter were then obtained in a follow-up interview. The data were then collated in a Microsoft Excel spreadsheet, in which tables were constructed, and parameters such as means, percentages and standard deviations calculated. Chi square was calculated using interactive chi square software.^20^ A *p*-value of less than 0.05 was considered significant.

## Results

Of the 300 sampled subjects, 210 (70%) were fish smokers. None of the fish smokers smoked cigarettes, and none declined to participate in the study. Their mean age was 31.46 ± 13.03 years (Table 1).

**TABLE 1:** Age distribution of fish smokers.

Age range (yeas)	Fish smokers	

	*n*	%
15–24	84	39.8
25–34	47	22.6
35–44	33	15.7
45–54	31	14.9
55–64	15	6.9

**Total**	**210**	**100**

*n*, number of fish smokers.

The 15-24 age group contained the largest percentage (39.8%) of subjects, with the smallest percentage (6.9%) occurring in the 55-64 age group. The prevalence of respiratory symptoms amongst the subjects was found to be 86.7%, which increased with age, with a dip to 83.2% in the 35-44 age group. Such prevalence was found to be highest (94.7%) in the 55-64 age group (Table 2).

**TABLE 2:** Prevalence of respiratory symptoms in different age groups.

Age range (years)	Respiratory symptoms				Total (*N*)

	Present		None		
		
	*n*	%	*n*	%	
15–24	72	86.2	12	13.8	84
25–34	41	88.2	6	12.8	47
35–44	27	83.2	6	16.8	33
45–54	28	90.2	3	9.8	31
55–64	14	94.7	1	5.3	15

**Total**	**182**	**86.7**	**28**	**13.3**	**210**

*n*, number of fish smokers; *N*, total number of fish smokers with and without Respiratory symptoms.

The educational status of the subjects was found to be low, with only one participant (0.48%) having completed tertiary level education (Table 3).

**TABLE 3:** Distribution of the fish smokers, in terms of their educational level.

Educational level	Fish smokers	

	*n*	%
No formal education	87	41.42
Primary education	88	41.90
Secondary education	34	16.20
Tertiary education	1	0.48

**Total**	**210**	**100**

*n*, number of fish smokers.

The most prevalent respiratory symptoms amongst the subjects were catarrh (30.48%) and a cough (28.57%), whereas the least prevalent respiratory symptom was breathlessness (2.38%; Table 4).

**TABLE 4:** Prevalence of respiratory symptoms amongst fish smokers.

Symptoms	Fish smokers	

	*f*†	%
Sneezing	53	25.24
Catarrh	64	30.48
Cough	60	28.57
Phlegm	16	7.62
Breathlessness	5	2.38
Chest pain	49	23.33

*f*, frequency

† Total more than 210 because of multiple responses.

The symptom most commonly experienced during fish smoking was sneezing, from which 153 (73.0%) of the subjects suffered, whereas breathlessness occurred the least, in only 7 (3.3%) of the subjects. Sneezing stopped in 135 (64.2%) of the subjects after fish smoking ceased, whereas breathlessness improved in only 7 (3.3%) of the fish smokers surveyed. The difference in occurrence of the symptoms during and after fish smoking was not statistically significant (Table 5).

**TABLE 5:** Distribution of respiratory symptoms amongst subjects during and after fish smoking.

Respiratory symptoms	Smoking				*χ*^2^	*p*-value

	During		After			
		
	*n*	%	*n*	%		
Sneezing	153	73	135	64.2	1.13	0.29*
Catarrh	159	75.5	134	63.5	2.13	0.14*
Cough	138	66.1	114	54.4	2.29	0.13*
Phlegm	28	13.5	23	10.9	0.49	0.48*
Breathlessness	7	3.3	6	2.7	0.77	0.78*
Chest pain	59	28.1	46	21.9	2.86	0.09*

*n*, number of fish smokers

* Not statistically significant.

Of the 210 fish smokers studied, 205 (97.6%) smoked their fish indoors, whereas only 5 (2.4%) of the subjects smoked their fish outdoors.

## Discussion

Biomass incineration is the largest domestic energy source in developing countries.^2^ The availability and affordability of this form of energy makes it the preferred energy source for those who live in the rural areas of such countries, despite its negative health impact. The findings in the study population, which consisted only of women, were similar to findings which have been made in other parts of the world, where women are responsible for most of the cooking. As a result, both the women and their children, who spend a great deal of time together, tend to suffer from the effect of the inhalation of wood smoke.^2^ In the population studied, the men spend most of their time fishing at sea, whereas the women are responsible for the drying and marketing of fishing-related products. In the settlement surveyed, fish smoking is without doubt the only occupation which is readily available to young women, who are likely to have acquired the skill during their childhood, when they would have assisted their mothers with their smoking activities.

All the subjects in this study ranged between 15 years and 64 years of age, making their demographics similar to those of the subjects in the research conducted by Gregg and Nunn.^21^ With increasing age, fewer subjects were found to be involved with fish smoking, most probably because of the tedious nature of the activity and the adverse effects of fish smoking. These effects, generally, cannot be tolerated by the aged and might be a contributing factor to the low life expectancy encountered in the area.^22^ That the highest percentage (22.3%) of the participants belonged to the 15-24 age group further confirms the rigorous nature of the fish smoking, which requires physical fitness. Such a finding is at variance with that of Peters et al.,^15^ who found that most of the women involved in fish smoking were aged 40+ years. The socio-cultural setting of their study might, however, have accounted for such a difference, as well as the fact that their participants included both men and women.

The Oyorokoto settlement, which is a relatively isolated rural community, with little or no presence of government and other work providers, is unattractive to the educated classes of Nigerian society. The lack of well-paid job opportunities is a possible reason for most of the subjects in this study having a low level of education and the absence of schools in the area might also account for this phenomenon. The parents might not be willing to send their children to schools far from their homes, as they would not be available to assist with fish smoking. The gender disparity, which is clearly evident in the society, might also lead to few girl children receiving more than a rudimentary education, at best. Government intervention in the area is needed in the provision of social services that would improve the lot of those children who are in need of better education.

The high prevalence of respiratory symptoms amongst the fish smokers in this study is similar to findings by Rothman *et al*.,^23^ Ige and Onadeko,^24^ Nikolaus et al.^25^ and Ellegard^26^ whose subjects were exposed to wood smoke, charcoal smoke, kerosene and liquefied petroleum gas (LPG) smoke. These researchers reported the harmful effects of wood smoke on the respiratory system, compared to the effects of exposure to milder kerosene, LPG and electric fire. Biomass burning, either from indoor or outdoor combustion, has been found to emit toxic pollutants containing particulate matter, which is predominantly (94%) comprised of fine and ultra-fine particles. These particles penetrate deeply into the respiratory system, passing through the epithelial barrier and reaching the interstitial lung, where they trigger an inflammatory process.^27^

The most frequently occurring respiratory symptoms present during fish smoking were those of catarrh and sneezing. Whereas such symptoms were found, in most cases, to stop upon the cessation of fish smoking, breathlessness encountered by the subjects seemed to be ongoing, providing possible evidence of chronic obstructive airways disease (COAD). This finding was similar to that of Ige and Onadeko^24^ amongst saw millers, and by Nikolaus^25^ amongst charcoal workers. Women who have been exposed to wood smoke have been found to have five times greater risk of chronic bronchitis, in comparison with the risk suffered by those who are not exposed to wood smoke.^26^ The risk reported by Harris-Eze,^11^ Bruce et al.,^28^ Mishra,^29^ Ray,^30^ Metinkaya et al.^31^ and Jindal^32^ as being prevalent in these, and similar occupational settings, has been found to increase with the amount of indoor air pollution to which the subjects have been exposed.

Even though few of the subjects in this study smoked their fish outdoors, the direct effects of this on humans − in comparison with the effects of indoor fish smoking on humans − were found to be similar, though milder. These effects, including bronchial asthma, bronchitis, and ARIs, have been recorded in Malaysian, Indonesian and Singaporean populations during episodes of bush burning, in which the number of patients presenting with these symptoms in hospitals greatly increased.^33, 34^ Bush burning has also been found to contribute to the depletion of the ozone layer, with a resultant adverse effect on human health, plant growth, photosynthesis, the flowering of plants, and agricultural yields.^35, 36^

## Conclusion and recommendations

Our research has shown that some respiratory symptoms are associated with exposure to wood smoke. As a result, there is a need to introduce appropriate safety measures and preventive interventions. This includes health education, which should encourage fish smokers to wear a face-mask, or use a cloth over the mouth and nose during fish smoking. The building and use of local ventilation systems (such as chimneys), the opening of doors of rooms in which fish is smoked, and the smoking of fish outdoors should be advocated. In addition, the Chorkor (modern) method of fish smoking should be popularised, as the use of this method carries fewer health risks than the traditional method, which is still used in the Oyorokoto fishing settlement. The regular health assessment of fish smokers should be instituted to help ensure an improved quality of life. In the long term, the effect of biomass burning on humans and the environment should be minimised by the adoption of methods of smoking which are friendlier both to humans and to the environment. A clarion call has been made for the Nigerian government to provide social services for the local inhabitants of Oyorokoto fishing settlement, including enhanced educational opportunities for the young, especially for girl children who have been neglected in the past. Furthermore, a study assessing the respiratory function of fish smokers is recommended.
